# Nature-based interventions: a systematic review of reviews

**DOI:** 10.3389/fpsyg.2025.1625294

**Published:** 2025-08-08

**Authors:** Branislav Kaleta, Stephen Campbell, Jimmy O’Keeffe, Jolanta Burke

**Affiliations:** ^1^Centre for Positive Health Sciences, RCSI University of Medicine and Health Sciences, Dublin, Ireland; ^2^School of History and Geography, Dublin City University, Dublin, Ireland

**Keywords:** nature-based interventions, green space interventions, blue space interventions, horticulture, forest bathing, nature

## Abstract

**Background:**

Nature-based interventions are emerging as an alternative to therapeutic approaches aimed to reduce and prevent mental and physical ailments. However, little is known of the types of interventions available to use by healthcare professionals. This systematic review of reviews aimed to classify and categorise different types of Nature-Based Interventions (NBIs) which currently exist under different names and approaches. The second aim of our review was to explore the mediating and moderating factors impacting NBI effectiveness.

**Methods:**

The systematic review used the narrative synthesis approach following the PRISMA guidelines, using the following databases: Academic Search Complete, APA PsycInfo, CINAHL, MEDLINE, and included only peer-reviewed review articles in English which explored Nature Based Interventions (NBIs), excluding animal-based interventions. The quality review was conducted using AMSTAR-2.

**Results:**

The review included a total of 61 reviews of NBIs, covering 13 different categories of NBIs: nature-based interventions, horticulture, nature exposure, green exercise, wilderness and adventure therapy, forest therapy, blue space interventions, care farming, nature play, nature-based education, environmental volunteerism, immersive nature experiences, and caring for country. Furthermore, 11 moderating and mediating factors influencing NBI effectiveness were identified: social, physical activity, age, nature connectedness, duration and frequency, gender, symptom severity, environment type, participant motivation and preference, challenge confrontation, and autonomy, responsibility, and skill and knowledge acquisition.

**Conclusion:**

The current review found a wide variety of NBIs, showcasing the many different options available to individuals and healthcare professionals offering accessible and cost-effective NBIs. Moreover, the moderating and mediating factors identified in our review will help future researchers, healthcare professionals, and practitioners consider these factors when evaluating the effectiveness of NBIs.

**Systematic review registration:**

PROSPERO (https://www.crd.york.ac.uk/PROSPERO/view/CRD42023491598), identifier (CRD42023491598).

## 1 Introduction

Nature-based interventions (NBIs) are activities, programmes, or strategies which aim to improve a person’s mental and physical health by involvement in a nature-based experience (Shanahan et al., 2019). Among many other benefits, NBIs offer cost-effective ways of improving peoples’ physical and mental health and wellbeing (Hinde et al., 2021; Pretty and Barton, 2020). These cost-effective interventions are especially crucial for a world tackling increasing healthcare costs (OECD, 2024).

A wide variety of NBIs exist under different names, approaches, and target populations, many of which overlap. NBIs can refer to interventions which are both outdoors (Struthers et al., 2024) and indoors (Yeo et al., 2020), natural or virtual (Wen et al., 2024). In one specific example, forest therapy, forest bathing, or shinrin-yoku refer to immersing yourself within the forest using our five core senses (Wen et al., 2019). On the other hand, research on forest bathing using virtual reality is in development (Masters et al., 2024), although the use of all our five core senses in virtual reality is still severely limited. As such, this nomenclature calls into question whether virtual forest bathing is closer to watching videos of nature (Mauldin et al., 2025) than it is to forest bathing in a real forest. As such, there is a clear need for categorising different nature-based interventions.

Moreover, factors such as nature connectedness (Rosa et al., 2023), physical activity (Vella-Brodrick and Gilowska, 2022), or intervention duration (Yeo et al., 2020) may influence NBI effectiveness, yet they are sparsely controlled for or reported in individual NBI studies. A review on nature prescribing even found that evaluations of nature prescribing programmes was lacking (Kondo et al., 2020). Awareness of factors associated with NBIs and subsequent reporting on those factors in studies would facilitate comparisons between different studies. A more comprehensive synthesis of these factors would thus improve our understanding of the underlying mechanisms of NBIs and allow future studies, reviews, and meta-analyses to account for these factors and even aid in evaluating nature prescribing programmes.

Lastly, NBIs should offer individuals a range of options to choose from so that individuals can choose the NBI which works best for their surrounding environment and personal preferences. Some individuals may not be able to access green space, while others might not have access to a clean blue space. Personalisation is a well-known and effective strategy of improving therapy outcomes (Nye et al., 2023), and providing a wide spectrum of NBI options would be especially valuable for nature prescriptions, potentially improving patients’ engagement and health outcomes.

Taking things together, the research questions of this review are two-fold: Firstly, what are the different types and categories of NBIs? Secondly, what are the moderating and mediating factors associated with NBIs? By answering these two questions, we aim to clarify the NBI literature and provide a pathway towards improving research on NBIs and personalising NBIs according to individual preferences.

## 2 Methods

This review followed the PRISMA 2020 review guidelines (Page et al., 2021) and was registered with PROSPERO (CRD42023491598) ahead of the review.

### 2.1 Search strategy and inclusion and exclusion criteria

Electronic databases (MEDLINE, Academic Search Complete, APA PsycInfo, CINAHL Ultimate) and grey literature (Google Scholar, Semantic Scholar) were searched in February 2024 and further electronic databases (CINAHL Ultimate, MEDLINE, CINAHL Plus with Full Text, APA PsycInfo) were subsequently searched in January 2025. The January 2025 search was restricted to peer-reviewed systematic reviews in English and excluded animal-based interventions. The detailed list of keywords can be found below and the PICO framework can be found in Table 1.

**Table 1:** PICO framework for this review.

Population	Intervention	Control	Outcome
Inclusive of healthy and clinical populations	Nature-based interventions, excluding animal-based interventions	Any controls considered, including reviews considering studies with no controls	Any outcomes assessed

#### 2.1.1 Keywords

1. **Nature-Based Interventions**: blue gym* OR care farm* OR care-farm* OR eco therap* OR ecotherap* OR eco-therap* OR environmental volunteer* OR farm therap* OR farm-therap* OR forest bath* OR forest therap* OR forest* therap* OR forest-bath* OR garden prescr* OR garden therap* OR green care OR green exercise OR green gym* OR green prescri* OR healing garden* OR horticultural therap* OR horticulture therap* OR nature assisted therap* OR nature based rehabilitation OR nature intervention* OR nature play OR nature prescri* OR nature rehabilitation OR nature therap* OR nature view* OR nature-assisted therapy OR nature-based OR nature-based intervention* OR nature-based rehabilitation OR nature-based* OR NBI* OR outdoor exercise OR park prescri* OR rehabilitation garden* OR shinrin yoku OR social farm* OR social horticult* OR therap* farm* OR therap* garden* OR therap* horticult* OR wild play OR wilderness therap* OR wilderness-therap*.
2. **NOT:** narrow band imaging.
3. **Reviews:** review* OR systematic review*.

The search was restricted to only systematic reviews of the literature. As this review of reviews is focused on what types of NBIs exist, there were also no restrictions in terms of the types of outcomes.

### 2.2 Screening and extraction

References were downloaded from the databases and uploaded to Rayyan for de-duplication and screening. Review screening and selection were conducted by two researchers whereas data extraction was conducted by the main author. Disagreements over the inclusion and exclusion of reviews during review screening and selection were resolved in regular meetings and agreed upon by both researchers.

### 2.3 Quality review

The quality review was conducted by the main author using the online checklist version of the AMSTAR-2 (Shea et al., 2017), which categorises systematic review quality according to the number of critical and non-critical flaws and weaknesses. The quality review identified 3 high quality reviews, 13 moderate quality reviews, 18 low quality reviews, and 27 critically low quality reviews. There was an overall improvement in review quality over time. All reviews were considered for exploring the different types of NBIs as the review quality does not impact the type of NBI it explored. Moreover, this approach is appropriate especially for exploring less common NBIs which might be underrepresented in the literature. Regarding the moderating and mediating factors, only moderate and high quality reviews were considered to include only factors with strong supporting evidence.

### 2.4 Data synthesis

The review used the narrative synthesis approach (Popay et al., 2006) to best describe and summarise the findings of our review.

## 3 Results

**Figure 1:**
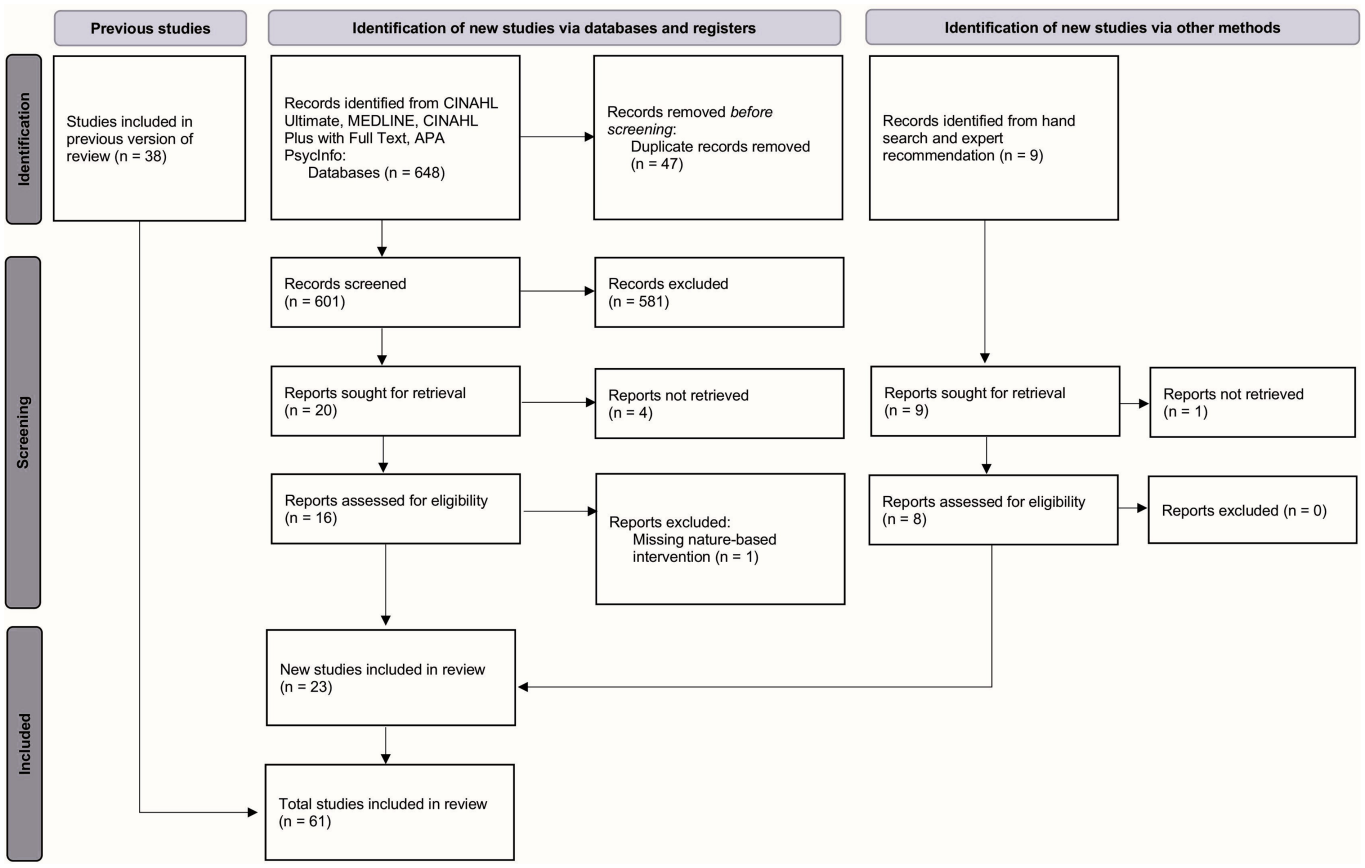
PRISMA 2020 flow diagram of the systematic review process.

The details of the literature search and screening process can be found in Figure 1.

### 3.1 Data extraction

The results of the data extraction can be found in Supplementary Table 1. The table contains the following information from the studies included in the review: Review, Population, Intervention, Control, Outcome, Moderating/Mediating Factors, and Conflict of Interest and Funding.

### 3.2 NBI types

The types of NBIs in the literature were identified and grouped according to thematic and conceptual similarities. Sixteen reviews reviewed horticultural interventions (Annerstedt and Währborg, 2011; Atchison et al., 2024; Bikomeye et al., 2022; Genter et al., 2015; Giang et al., 2024; Kondo et al., 2018; Lee et al., 2024; Lu et al., 2019; Mmako et al., 2020; Rueff and Reese, 2023; Trøstrup et al., 2019; Walker-Mao et al., 2024; Wang M. et al., 2024; Wang et al., 2022; Yun et al., 2024; Zhao et al., 2022), twelve reviews on nature exposure and nature viewing (Bikomeye et al., 2022; Corazon et al., 2019; Djernis et al., 2019; Fan and Baharum, 2024; Kondo et al., 2018; Paredes-Céspedes et al., 2024; Vella-Brodrick and Gilowska, 2022; Walker-Mao et al., 2024; Wang Y. et al., 2024; Wen et al., 2024; Yeo et al., 2020; Zhao et al., 2022), ten reviews on green exercise (Bikomeye et al., 2022; Coventry et al., 2021; Fraser et al., 2020; Kondo et al., 2018; Ma et al., 2023; Mmako et al., 2020; Paredes-Céspedes et al., 2024; Picton et al., 2020; Rueff and Reese, 2023; Struthers et al., 2024), ten reviews on Wilderness/Adventure Therapy (Annerstedt and Währborg, 2011; Bettmann et al., 2016; Bowen and Neill, 2013; Fatima et al., 2022; Gillis et al., 2016; Kraft and Cornelius-White, 2020; Pomfret et al., 2023; Rosa et al., 2023; Rueff and Reese, 2023; Shanahan et al., 2009), ten reviews on forest bathing (Bikomeye et al., 2022; Caponnetto et al., 2022; Fatima et al., 2022; Kamioka et al., 2012; Kotera et al., 2022; Lee et al., 2017; Quan et al., 2020; Rueff and Reese, 2023; Siah et al., 2023; Wen et al., 2019), five on NBIs (Fatima et al., 2022; Gritzka et al., 2020; Obeng et al., 2023; Taylor et al., 2022; Trøstrup et al., 2019), four on blue space interventions (Britton et al., 2020; Carneiro et al., 2024; Guntur et al., 2023; Overbury et al., 2023), four on nature-assisted therapy (Annerstedt and Währborg, 2011; Kotera et al., 2022; Rueff and Reese, 2023; Smith et al., 2024), three on ecotherapy (Caddick and Smith, 2014; Fatima et al., 2022; Rueff and Reese, 2023), two on farming (Cano-Verdugo et al., 2024; Gorman and Cacciatore, 2017), two on nature play (Dankiw et al., 2020; Johnstone et al., 2022), two on nature-based education (Ly and Vella-Brodrick, 2024; Miller et al., 2021), one on environmental volunteerism (Chen et al., 2022), one on immersive nature experiences (Mygind et al., 2019), and one about caring for country (Fatima et al., 2022).

#### 3.2.1 Nature-based interventions/eco-therapy/nature-assisted therapy

NBIs can be understood as an overall umbrella term for any of the types of NBIs in this review. Similarly, eco-therapy can also be used as an overarching term for both active (exercise, gardening) and passive (nature exposure) nature-based techniques and practices. Nature-Assisted Therapy can also be understood as a broad term covering such practices, with the aim of recovering a patient’s health using interventions involving plants, natural materials, or outdoor environments (Annerstedt and Währborg, 2011).

#### 3.2.2 Horticulture/horticultural therapy

Horticulture-based interventions are the most common type of interventions appearing in the reviews of this review. The activities in these interventions mainly involve gardening and other forms of taking care of plants (Tu, 2022).

#### 3.2.3 Nature exposure/nature viewing

Nature exposure interventions typically involve exposure to outdoor nature, but can also include interventions using simulated nature indoors (Yeo et al., 2020). It is important to differentiate between outdoor nature exposure interventions and green exercise and wilderness therapy as nature exposure and nature viewing interventions should be more passive and at most include a light level of activity, as physical activity can have effects beyond nature exposure itself (Corazon et al., 2019; Kondo et al., 2018). For example, one review claimed forest therapy to involve nature viewing (Kondo et al., 2018).

#### 3.2.4 Green exercise

Green exercise interventions consist of engaging in physical activity, such as walking or running, in nature (Bikomeye et al., 2022). These interventions are different from wilderness/adventure therapy in that although they do involve physical activity, their focus is not on overcoming challenges.

#### 3.2.5 Wilderness/adventure therapy

The key features of Wilderness/Adventure Therapy Programmes usually involve some form of more complex, longer-term outdoor exercise activities hiking, trekking, and camping from 2 weeks to as long as 3 months (Bettmann et al., 2016). Likewise, the core of adventure interventions involves a certain form of risk in nature, such as camping, backpacking, or skiing. As such, these interventions can encompass both wilderness and adventure intervention programmes (Rosa et al., 2023).

#### 3.2.6 Forest bathing/forest therapy

Forest bathing, also called shinrin-yoku, is a practice involving conscious and mindful immersion in the forest through all five senses. Forest therapy is related to forest bathing in the sense that it is a clinical application of forest bathing with a focus on specific physical and mental health difficulties (Caponnetto et al., 2022).

#### 3.2.7 Blue space interventions

Most of the other NBIs mentioned cover green spaces, but blue spaces are just as viable for NBIs. Blue spaces can be defined as all visible, outdoor, natural surface waters with the potential to promote human health and wellbeing (Britton et al., 2020). As such, as of right now, all interventions involving blue spaces would be under the umbrella term Blue Space Interventions, and such interventions may include surfing (Carneiro et al., 2024), scuba diving (Guntur et al., 2023), or outdoor swimming (Overbury et al., 2023). Although there is some overlap between blue and green spaces, some reviews argue that blue spaces can offer original benefits which might be inaccessible in green spaces alone (Britton et al., 2020).

#### 3.2.8 Care farming

Care farming involves the use of farms and agricultural landscapes for promoting mental and physical health through regular farming activities (Gorman and Cacciatore, 2017).

#### 3.2.9 Nature play

Nature play interventions are nature-based, unstructured interventions aimed at children which allow them to play in environments containing natural elements such as gardens, forests, ponds, water, mud, plants, or rocks (Dankiw et al., 2020).

#### 3.2.10 Nature-based education

Nature-Based Education or Nature-Based Learning is an approach in education which utilises the natural environment to facilitate learning (Miller et al., 2021). Such activities can include simply using a natural area as an outdoor classroom to actively using the natural environment in classes.

#### 3.2.11 Environmental volunteerism

Environmental volunteerism involves activities such as tidying trails, soil preparation, tree planting, or recycling (Chen et al., 2022) and whereas most of the aforementioned interventions used nature in some way to improve mental and physical health, environmental volunteerism gives back to nature and Earth to improve our health.

#### 3.2.12 Immersive nature experiences

Also called “friluftsliv,” this Scandinavian tradition covers a wider range of interventions: outdoor life, outdoor recreation and education, or adventure recreation and education, with an emphasis on achieving closeness to nature (Mygind et al., 2019).

#### 3.2.13 Caring for country

Caring for Country is less of an intervention and more of a tradition for Indigenous Australians. It involves spending time in the country, revegetation, harvesting, protecting sacred areas and threatened species, or controlling fires, weeds, or feral animals (Fatima et al., 2022). These activities express the deeply interconnected relationship between the people and the country, from which both the people and the country benefit. These activities may resemble environmental volunteerism, as perhaps environmental volunteerism is a way for our modern population to try to reconnect and care for their country again.

#### 3.2.14 Active and passive interventions and focus on nature

Two types of interventions have emerged from the analysis. The first type explored the level of activity required for participants to engage with NBIs. Active interventions were characterised by the participants’ direct physical, behavioural, or cognitive engagement. These interventions included such activities as movement, decision-making, skill-building, or overcoming challenges that require sustained and intentional participation. In contrast, passive interventions referred to experiences in which individuals receive the benefits of nature with minimal physical or mental effort. These were less demanding and included such activities as simply being present in a natural environment, viewing nature, or engaging in undirected relaxation outdoors. The terms Nature-Based Interventions and Blue Space Interventions were excluded as they cover a wide range of interventions including both passive and active ones, making them unfit for this distinction. For more details on this active and passive distinction for each NBI please see Table 2.

**Table 2:** Passive and active type of NBIs with a rationale for inclusion.

NBI type	Passive/Active	Rationale
Horticultural therapy	Active	Involves gardening and plant care, thus requires physical engagement
Nature exposure/viewing	Passive	Requires minimal engagement as it involves sitting or walking slowly while observing nature
Green exercise	Active	Involves physical activity in nature, such as walking, running and other intentional movement
Wilderness/Adventure therapy	Active	Includes extended, challenges outdoors requiring intense participant involvement
Forest bathing	Passive	The focus is on mindful presence and sensory immersion so it can involve slow movement, but it does not require intense physical engagement
Care farming	Active	Engages participants in farm work, thus requires physical engagement
Nature play	Active	Involves unstructured physical and imaginative activity in nature
Nature-based education	Active	Includes learning through doing in a natural setting, thus it could be physically, cognitively and emotionally engaging
Environmental volunteerism	Active	Involves physically demanding activities, such as planting or cleaning
Immersive nature experiences	Both	Includes recreation and education outdoors and can involve either passive or active recreation
Caring for country	Active	Involves culturally-driven practices that include interaction with land and environment

The second type of NBI that has emerged refers to whether nature was a primary or secondary focus. In NBIs that viewed nature as a primary focus, nature was the central therapeutic or educational agent, and the benefits of NBIs could be easily linked to nature. These NBIs included activities such as forest bathing, where the primary focus is on connecting with nature, or horticultural therapy, which focuses on attending to nature. On the other hand, in NBIs that viewed nature as a secondary focus, nature played a supportive or contextual role. These NBIs included outdoor classrooms, where the learning experience was the primary focus or green exercise, where physical activity is the primary focus of an activity, and nature is the context within which it is conducted. Similarly to the previous table, the terms Nature-Based Interventions and Blue Space Interventions were excluded as they cover a wide range of interventions. For more details on this primary and secondary distinction for each NBI please see Table 3.

**Table 3:** NBIs where nature is seen as a primary or secondary focus.

NBI type	Nature as a primary or secondary focus	Rationale
Horticultural therapy	Primary	Involves noticing the beauty of nature and creating it
Nature exposure/viewing	Primary	Nature is the main reason for engaging in this activity
Green exercise	Secondary	Physical activity is the primary reason
Wilderness/Adventure therapy	Secondary	Challenges are the main reason for this activity
Forest bathing	Primary	Nature is the therapeutic agent and the activity helps individuals connect with nature
Care farming	Secondary	Farming is the main focus
Nature play	Secondary	Play is the main focus and nature is contextual
Nature-based education	Secondary	Education is the main focus
Environmental volunteerism	Secondary	Volunteerism is the main focus
Immersive nature experiences	Primary	Nature is the primary focus of the experience, its beauty and engagement with it
Caring for country	Secondary	It is about the relationship with the land and restoring it

### 3.3 NBI moderating/mediating factors

The review identified multiple possible moderating/mediating factors potentially impacting the effectiveness of a given NBI. Of the 16 high quality and moderate quality reviews, only 7 found significant moderating/mediating factors. These factors were: social (Overbury et al., 2023; Rosa et al., 2023; Vella-Brodrick and Gilowska, 2022; Yeo et al., 2020), physical activity (Overbury et al., 2023; Rosa et al., 2023; Vella-Brodrick and Gilowska, 2022), age (Ly and Vella-Brodrick, 2024; Vella-Brodrick and Gilowska, 2022), nature connectedness (Overbury et al., 2023; Rosa et al., 2023), duration and frequency (Lee et al., 2024; Vella-Brodrick and Gilowska, 2022; Wang M. et al., 2024; Wang Y. et al., 2024; Yeo et al., 2020), gender (Ly and Vella-Brodrick, 2024), symptom severity (Lee et al., 2024), environment type (Wang M. et al., 2024), participant motivation (Rosa et al., 2023), challenge confrontation (Rosa et al., 2023), dementia (Yeo et al., 2020), skill/knowledge acquisition (Yeo et al., 2020), and autonomy/responsibility (Yeo et al., 2020).

#### 3.3.1 Social

The social factor appeared in four out of five reviews discussing the moderating/mediating factors. In one study, a two-year exposure to a schoolyard improved children’s social well-being (Vella-Brodrick and Gilowska, 2022). However, social interaction was also an important aspect of NBIs for older adults (Yeo et al., 2020). Moreover, positive social interactions may improve hopelessness and feeling bad about oneself in depression (Rosa et al., 2023).

#### 3.3.2 Physical activity

Many of the NBIs in this review involve some form of physical activity, which may be another factor in terms of many of the benefits of NBIs (Rosa et al., 2023; Vella-Brodrick and Gilowska, 2022), as physical activity and the outdoors are inherently linked.

#### 3.3.3 Age

In one review, nature exposure did not seem to have the same effects on school children aged 7–12 when compared to middle or high school students (Vella-Brodrick and Gilowska, 2022). Another review found mixed effects of age in relation to mental, physical, and social wellbeing (Ly and Vella-Brodrick, 2024).

#### 3.3.4 Nature connectedness

As some NBIs can strengthen a participant’s nature connectedness (Rosa et al., 2023), or in the case of blue space interventions, water connectedness (Overbury et al., 2023), a person’s nature connectedness might impact how responsive they are to a given NBI.

#### 3.3.5 Duration and frequency

In studies with older adults, a larger proportion of the studies which lasted more than 5 weeks (7 out of 9) reported significant findings compared to the proportion of studies lasting 5 weeks or less (3 out of 9) (Yeo et al., 2020). Another review also reported the benefits of NBIs on attentional functioning in children in the short term, while the benefits of long-term NBIs were difficult to assess (Vella-Brodrick and Gilowska, 2022). As the former review focused on older adults and the latter on children and cognitive benefits, the efficiency of short-term vs. long-term NBIs is difficult to disentangle. As for frequency, horticultural interventions of 2 or more in frequency were more beneficial (Wang et al., 2024).

#### 3.3.6 Gender

One review found differences between the effects of gender on physical activity between boys and girls at school (Ly and Vella-Brodrick, 2024), where although boys were found to have more physical activity overall, it was girls’ physical activity which benefited most from NBIs.

#### 3.3.7 Symptom severity

One review on horticultural therapy and individuals with schizophrenia (Lee et al., 2024) found that symptom severity was important, as the effect sizes were larger for those with moderate severity compared to those with mild severity.

#### 3.3.8 Environment type

A review on horticultural therapy in older patients with dementia (Wang M. et al., 2024) found outdoor interventions to have a larger effect size than indoor interventions.

#### 3.3.9 Participant motivation and preference

One review highlights the importance of participant motivation and preference on the effectiveness of NBIs (Rosa et al., 2023). The more the participants are motivated to participate in the intervention, and the more the intervention aligns with the participants’ preferences, the less likely they may be to drop out of the intervention.

#### 3.3.10 Challenge confrontation

Overcoming a challenge, which is an aspect present in some adventure therapies, has been found to be an effective way of improving the symptoms of depression by potentially improving the participants’ self-confidence and resilience (Rosa et al., 2023).

#### 3.3.11 Autonomy, responsibility and skill and knowledge acquisition

NBIs which involved providing older adults with autonomy, responsibility, and some form of skill or knowledge acquisition, were more likely to be effective (Yeo et al., 2020). Another review shares a similar view, noting that the satisfaction of needs such as autonomy, competence, and relatedness might partly explain the mental health benefits of NBIs (Rosa et al., 2023). As such, an improved perception of autonomy through NBIs might improve their effectiveness. The acquisition of new knowledge and skills in older adults can thus not only improve the effectiveness of NBIs but also improve their lives.

## 4 Discussion

The aim of this systematic review of reviews was two-fold: Map out what types of NBIs exist and find out what moderating or mediating factors might be impacting their effectiveness. The review found a wide spectrum of NBIs discussed in the literature and multiple factors potentially impacting their effectiveness.

Our findings show a wide spectrum of NBIs offering unique mechanisms for improving peoples’ mental, physical, and social wellbeing, combined with a combination of multiple core factors influencing their effectiveness. The benefits of these interventions are well-established in the literature covered in our review, and our analysis highlights the need to tailor interventions to individual characteristics. For example, while horticultural therapy is frequently employed due to its robust evidence base for mental health improvements (Tu, 2022), interventions like environmental volunteerism, though primarily focused on older adults (Chen et al., 2022), also benefit younger populations (McDougle et al., 2011). This underscores the need for identifying the right person-intervention fit in future research.

The correct person-intervention fit may relate to the type of interventions used. The current review identified two types of interventions, i.e., those that are passive vs. active and interventions where nature is a primary focus vs. a secondary focus. These distinctions are important for several reasons. First, they help clarify the mechanisms of change, making it more effective to apply behavioural change models. For example, one of the most prevalent models is the COM-B model of change (Michie et al., 2011), which consists of three elements: capability (skill-building, support in knowing NBIs), opportunity (accessibility of green spaces and tools), and motivation (goal-setting, habit-formation). The opportunity aspect of COM-B may be especially relevant to nature-based interventions and urban planning, as better access to and improved quality of green and blue spaces are associated with improved physical and mental health benefits (Ekkel and de Vries, 2017).

Passive interventions may be particularly suitable for those who have low capability or limited access to quality nature. Active NBIs offer greater physical and psychological benefits but require more motivation to engage. Similarly, NBIs that have nature as a secondary focus might be easier to implement than those less familiar or comfortable with engaging with NBIs. Primary NBIs, on the other hand, require a stronger alignment across all three components of the COM-B model, but may also result in deeper nature-connectedness. Thus, distinguishing between the different types of interventions can be useful in clarifying the mechanisms for change. Subsequently, they will help tailor interventions to individuals’ needs, improving the impact and sustainability of NBIs. Further research needs to explore the differences in the impact of these interventions through a meta-analysis and establish in what situations and with whom they would be most effective.

### 4.1 NBI factors

The factors impacting NBI effectiveness are a complex, interconnected matrix of characteristics of given NBIs and people participating in them. As such, it is essential to consider that these factors influence not only NBI effectiveness but also each other. Identifying similarities and inconsistencies between these factors can help researchers and practitioners design NBI approaches that maximise the impact of nature on their wellbeing.

#### 4.1.1 Social

This overall pattern of the importance of the social aspects of NBIs is difficult to ignore. NBIs can not only bring people together, but also improve people’s social skills (Bloomfield, 2017). As such, social NBIs might be effective at combating increasing loneliness (Buecker et al., 2021), and implementing group NBIs might bring about greater benefits than individual ones.

#### 4.1.2 Physical activity

Nature can not only make it easier for people to achieve sufficient levels of physical activity, it can also enhance the amount and intensity of the physical activity itself (Gladwell et al., 2013). Researchers exploring NBIs involving physical activity should account for the benefits of physical activity and ensure the benefits are not purely based on the physical activity involved in the NBI. Similarly, practitioners may find it useful to account for patients’ physical ability when choosing appropriate NBIs and recommending NBIs involving physical activity when suitable.

#### 4.1.3 Age

The reviews included in our review cover all age ranges from children to older adults, and as such, NBIs appear to be appropriate for individuals of any age. However, age remains a factor (Shanahan et al., 2019), as not all NBIs are equally appropriate for all ages. For example, more physically intensive NBIs such as weeks or months-long hikes might be too difficult, if not detrimental, to older adults. Future research should thus take into consideration the differences in the effectiveness of NBIs between major age groups and throughout childhood and adolescence. In addition, practitioners may want to take age into account especially in combination with the physical requirements of an NBI.

#### 4.1.4 Nature connectedness

Nature connectedness can change how individuals interact with nature (Martin et al., 2020). For example, blue space interventions such as swimming claim to involve more immersion in nature (Overbury et al., 2023) than green space interventions, as you are surrounded and within the water. The question is whether this complete connectedness of being within nature changes peoples’ nature connectedness differently than just looking at nature on a screen such as in nature viewing. Future studies may not only explore an individual’s nature connectedness before the study, they may also explore how a person’s nature connectedness changes throughout the different types of NBIs.

#### 4.1.5 Duration and frequency

NBIs may last anywhere from a few minutes, hours, to weeks or months. As such, it is essential to narrow down the minimum efficient “dose” of NBIs (Wilkie and Davinson, 2021) in terms of both duration and frequency. Future research should thus explore experiment duration and frequency as an essential variable to determine NBI efficiency. Ascertaining the minimum effective dose of NBIs would be of special benefit to healthcare professionals and practitioners.

#### 4.1.6 Symptom severity

Symptom severity has been found to be an important factor, which aligns with other mental health literature, for example, with depression severity and antidepressant effects (Fournier et al., 2010), with patients with increased severity seeing more benefit from antidepressants. As such, NBIs might be especially beneficial for people with more severe mental health symptomology.

#### 4.1.7 Environment type

Outdoor interventions have been found to be more effective than indoor ones, aligning with previous literature on exercise and how people find outdoor exercise to be more enjoyable than indoor exercise (Noseworthy et al., 2023). Healthcare practitioners and clinicians should thus consider outdoor NBIs before indoor ones.

#### 4.1.8 Participant motivation and preference

Treatment preference is associated with decreased rates of dropping out of treatments (Windle et al., 2020). Developing personalised NBIs and ensuring they are interventions people want to do themselves would thus increase the likelihood of them participating in the intervention and not dropping out.

#### 4.1.9 Challenge confrontation

Research shows confronting a challenge may lead to improvements in depression symptoms after NBIs (Rosa et al., 2023), however, this factor might have broader applicability. Improvements in peoples’ self-confidence and resilience might apply to non-depressed individuals as well (Liu et al., 2020). As such, when considering patients with impaired self-confidence, resilience, or depression, NBIs involving challenge confrontation might prove beneficial.

#### 4.1.10 Autonomy, responsibility and skill and knowledge acquisition

Autonomy refers to the extent to which a person can live an independent life and is often decreased in older adults (Sánchez-García et al., 2019). Learning new skills and knowledge is not just a way for older adults to spend time, it can also be a way for them to chase subjects and skills they did not have time to invest in during their life (Narushima, 2008), providing countless benefits. NBIs which improve a person’s autonomy and skills may thus be especially beneficial for older adults.

### 4.2 Strengths and limitations

One of the strengths of this review is that it covered a wide range of NBIs with different names and methodologies. Moreover, the review also identified less common NBIs such as environmental volunteerism, caring for country, and multiple blue space interventions. A limitation of this review is that it did not cover NBIs utilising animals, such as animal-assisted therapies (Kamioka et al., 2014). Another limitation of the review is that it only included peer-reviewed systematic reviews in English, potentially missing out on less common or newly researched NBIs and factors impacting them, and NBIs from different settings and non-English-speaking countries. Furthermore, as only 16 of the 61 reviews were of high enough quality to be considered for the mediating/moderating factor analysis, and only 7 of the reviews explored such factors, the current review might have missed out on some factors only included in the lower quality reviews. As this is a review of reviews and not a review of NBI studies, the review might have missed out on NBIs which have not been reviewed yet. For example, stargazing would certainly fall into the category of NBIs (Bell et al., 2014), however, due to limited literature on the benefits of stargazing and a lack of reviews including it, it wasn’t possible to include it in our review.

## 5 Conclusion

Our review set out to explore what types of NBIs exist and what factors may impact their effectiveness. The review found a wide variety of NBIs under different umbrella terms and names and attempted to differentiate between the found NBIs in a systematic way based on their features. Although the review found 13 different types of NBIs, it is essential to note that most of these NBIs overlap in terms of their features and aims. In terms of factors, this review found a spectrum of factors potentially impacting NBI efficacy. Future research can not only utilise the NBI terms categorised in this review, it can also consider the factors found in this review when researching and applying NBIs to improve people’s mental and physical health and overall wellbeing. Furthermore, healthcare professionals and policymakers may benefit from the findings of the review by considering the wide spectrum of NBIs available in order to bring personalised and cost-effective treatments into healthcare.

## Data Availability

The original contributions presented in the study are included in the article/Supplementary material, further inquiries can be directed to the corresponding authors.
