# Supplementary material for: Nature-based interventions: a systematic review of reviews
Source: Front Psychol. 2025 Aug 8;16:1625294. doi: 10.3389/fpsyg.2025.1625294 (PMC12370496; doi:10.3389/fpsyg.2025.1625294)
Supplement: Supplementary file 1 [file Table_1.docx]

**Supplementary Table 1.** *Data extraction table of reviews included in this review*

| **Review** | **Population** | **Intervention** | **Control** | **Outcome** | **Moderating / Mediating Factors** | **Conflict of Interest and Funding** |
| --- | --- | --- | --- | --- | --- | --- |
| (Annerstedt & Währborg, 2011) | No restriction | Horticultural Therapy, Wilderness Therapy, Nature-Assisted Therapy | No comparison, Relapse Prevention Programmes, Art Therapy, Cognitive Stimulation Games, Exercise, Craft Working, Current Events Discussions, Standard Care for Schizophrenia, Group Programmes, Relaxation Training Programmes, Aerobic Exercise | No restriction | N/A | Reported no specific grant from any funding agency in the public, commercial, or non-profit sector |
| (Atchison et al., 2024) | Older adults living in Long-Term Care | Horticultural Therapy | Randomised Control Trial | Depression Treatment Efficiency | None | Reported no specific funding or conflict of interest |
| (Bettmann et al., 2016) | No restriction | Wilderness Therapy, Adventure Therapy | No restriction | No restriction | N/A | Unreported |
| (Bikomeye et al., 2022) | No restriction | Forest Bathing, Green Exercise, Gardening, Nature Viewing | No restriction | Cardio-Vascular Disease, Cancer-Related Outcomes | N/A | Reported no conflict of interest The American Heart Association (AHA) Scientific focused research network (SFRN) on disparities in Cardio-oncology grants The AHA SFRN Research supplement to promote diversity in science The National Institutes of Health (NIH) grants The Medical College of Wisconsin Cancer Center grant The Medical College of Wisconsin Cardiovascular center grant ‘WeCareFund’ |
| (Bowen & Neill, 2013) | N/A | Adventure therapy | N/A | N/A | N/A | Reported no conflict of interest |
| (Britton et al., 2020) | No restriction | Blue Space Interventions | No restriction | Health, Wellbeing | N/A | Funded by the Environmental Protection Agency (EPA) and the Health Service Executive |
| (Caddick & Smith, 2014) | Veterans | Ecotherapy | No restriction | No restriction | N/A | Unreported |
| (Cano-Verdugo et al., 2024) | Any adults | Urban Farming | N/A | Health | None | Reported no specific funding or conflict of interest |
| (Caponnetto et al., 2022) | Patients with psychophysiological disorders from mild to severe, such as university stress, postmenopausal women, elderly, depression in alcoholics, people with chronic widespread pain, veterans with post-traumatic stress disorder (PTSD), and patients with affective and psychotic disorders | Forest Therapy | No restriction | Physiological and Psychological Well-Being | N/A | Reported no conflict of interest |
| (Carneiro et al., 2024) | Participants of any age, sex, explicitly diagnosed with any mental health disorder | Surf Therapy | Non-exercise or alternative interventions | Changes in mental health disorder symptoms | None | Reported no specific funding or conflict of interest |
| (Chen et al., 2022) | Older Adults | Environmental Volunteerism | No restriction | No restriction | N/A | Reported no conflict of interest |
| (Corazon et al., 2019) | Adults (18+), all ethnicities, all countries, with or without stress-related issues/illness, without mental disabilities or other serious physical or mental illness. | Exposure to all types of outdoor natural green environments, with all types of sedentary and light exercise activities, in all time durations. | All kinds of comparisons or no comparison group. | Physiological (cardiovascular, endocrine, and immune) and psychological (mood, physiological stress, and well-being) outcomes related to stress prevention or stress treatment. | N/A | Supported by the 15th of June Foundation, Denmark |
| (Coventry et al., 2021) | Adults | Nature-based outdoor activities for health (NOAH) | No restriction | Physical and Mental Wellbeing | N/A | Reported no conflict of interest UK Research and Innovation Closing the Gap Network+ |
| (Dankiw et al., 2020) | Children aged 2–12 years with no pre-existing/diagnosed physical, mental, behavioural or neurological health conditions | Unstructured Nature Play | No restriction | No restriction | None | Reported no specific funding or competing interests |
| (Djernis et al., 2019) | Adults and adolescents older than 12 | Nature Exposure  Nature-Based Mindfulness | Any | Psychological  Physiological  Interpersonal | No significant moderators, but explored type of nature and type of mindfulness | Reported no external funding and no conflict of interest |
| (Fan & Baharum, 2024) | People without audiovisual impairments | Nature Exposure | N/A | Stress reduction | None | Reported no specific funding or conflict of interest |
| (Fatima et al., 2022) | Indigenous People | Nature-Based Interventions, Caring for Country, Ecotherapy, Forest Therapy, Adventure Therapy | No restriction | Mental Health and Wellbeing | N/A | Reported no conflict of interest |
| (Fraser et al., 2020) | Adults diagnosed with type 2 diabetes mellitus. | Outdoor Activity, Green Exercise | No restriction | Change in psychological problems following the intervention (depression, anxiety, quality of life, emotional wellbeing and stress) | None | Reported no conflict of interest, funded by the European Social Fund |
| (Genter et al., 2015) | Adults | Allotment Gardening | No restriction | Health, Wellbeing | N/A | Reported no specific grant from any funding agency in the public, commercial, or not-for-profit sectors. |
| (Giang et al., 2024) | Older adults with normal cognition (Mean age ≥60), Mini Mental State Examination ≥23 or equivalent on validated cognitive assessment scales | Horticultural activities that involve passive or active interactions with plants | An active control group or an usual care group or a waitlist group that did not involve plant materials | Psychosocial outcomes Physical outcomes | None | Reported no conflict of interest Alexandra Health Fund Ltd |
| (Gillis et al., 2016) | 18 or younger | Wilderness therapy  Adventure therapy | N/A | N/A | N/A | Reported no conflict of interest |
| (Gorman & Cacciatore, 2017) | No restriction | Care Farming | No restriction | Trauma, Grief, Bereavement, Death, Mourning, Loss | N/A | Reported no specific grant from funding agencies in the public, commercial, or not-for-profit sectors. |
| (Gritzka et al., 2020) | Employees | Any NBI | Control group required | Any measurements of mental health and well-being assessed using questionnaire | N/A | Reported no conflict of interest, not funded by any specific grants |
| (Guntur et al., 2023) | People with physical disabilities | Scuba Diving | Controlled studies | N/A | None | Unreported |
| (Johnstone et al., 2022) | Children aged 2-7 years attending early childhood education | Early Childhood Education incorporating Nature | Early Childhood Education with no or little nature components | Physical Health, Development | N/A | Scottish Government’s Early Learning and Child Care Directorate UK Medical Research Council and Scottish Chief Scientific Officer Fundagao para a Ciencia e Tecnologia Scottish Funding Council |
| (Kamioka et al., 2012) | No restriction | Forest Therapy | No restriction | blood pressure, alpha brain wave amplitude, parasympathetic nervous activity, muscle tension, blood glucose level, NK activity, pain, and mental health status (mood, self-esteem, emotion, and subjective wellbeing or happiness) | N/A | Reported no conflict of interest |
| (Kondo et al., 2018) | No restriction | Nature Viewing, Outdoor Walks, Outdoor Exercise, Gardening | No restriction | Stress | N/A | Unreported |
| (Kotera et al., 2022) | Any population | Forest bathing  Nature Therapy | Any including no intervention | Mental Health | N/A | Reported no conflict of interest |
| (Kraft & Cornelius-White, 2020) | Adolescents | Wilderness Therapy | No restriction | No restriction | N/A | Unreported |
| (Lee et al., 2017) | Any adults | Forest therapy | At least one control group | Depression | N/A | Reported no conflict of interest  Korea Forest Service |
| (Lee et al., 2024) | Adult individuals diagnosed with schizophrenia | Horticultural Therapy | Randomised Control Trial | Symptoms Emotional States | Duration Symptom Severity | Reported no conflict of interest Kaohsiung Chang Gung Memorial Hospital Medical Education Department |
| (Lu et al., 2019) | Patients with Dementia/Alzheimer’s | Horticultural Therapy | No restriction | No restriction | N/A | The authors received no financial support for the research, authorship, and/or publication of this article. |
| (Ly & Vella‐Brodrick, 2024) | School-going children and adolescents aged 5–19 years | School-led nature (greenspace) exposure intervention | Urban, classroom, | Mental wellbeing: - Positive affect - Negative affect - Stress reduction/stress restoration Physical wellbeing: - Physical activity Social wellbeing: - Social relationships/interactions - Pro-social behaviour | Age Gender | Reported no specific funding or conflict of interest for the manuscript Open Access funding by Council of Australian University Librarians |
| (M. Wang et al., 2024) | Older Patients With Dementia | Horticultural Therapy | Standard Nursing Care | Cognitive Function, Depression Levels, Activities of Daily Living, Quality of Life | Duration Frequency  Environment type | Reported no specific funding or conflict of interest |
| (Ma et al., 2023) | Adults | Nature-based walking intervention, walking in green spaces, such as urban parks, farmland, forests, national parks, hills, landscape fields (including places such as golf courses) | Non-green outdoor spaces, such as urban area without significant greenery | Mental health was defined broadly to include measures such as: mood, mindfulness, anxiety, depression, subjective well-being, quality of life | N/A | Reported no conflict of interest |
| (Miller et al., 2021) | Primary school-aged children (age 4-12) | Nature-Based Learning, Nature Play | Traditional Classroom Learning | No restriction | None | Reported no conflict of interest Australian Government Research Training Program |
| (Mmako et al., 2020) | Patients with Dementia/Alzheimers/Mild Cognitive Impairment | Horticulture, Green Outdoor Activity | No restriction | No restriction | N/A | Reported no conflict of interest and no specific grant funding |
| (Mygind et al., 2019) | No restriction | Friluftsliv | Comparator groups exposed to urban or indoor settings. | Somatic Health, Mental Health, Social Health | N/A | Research funded by a grant from The Danish Outdoor Council, Denmark |
| (Obeng et al., 2023) | Young People | Programs that used nature either alone or as the main intervention along with other approaches such as therapy, labor market activation, or social rehabilitation to promote well-being Only considered NBIs that happened within the natural environment or outdoors | No restriction | Wellbeing | N/A | Reported no conflict of interest Natural Resources Institute Finland European Union’s Horizon 2020 Research and Innovation Program University of Jyväskylä |
| (Overbury et al., 2023) | Any human participant | Outdoor Swimming or Bathing | People who are not open-water swimmer groups, people who do other outdoor activities | Mental Health Wellbeing | Water Connectedness Social | Unreported |
| (Paredes-Céspedes et al., 2024) | Healthy adult participants | Interventions in natural settings, including forests, natural parks, urban parks, urban green areas, cultivated fields, or gardens, involving activities like walking, observing, engaging in relaxation exercises, or simply resting and breathing fresh air for a specified duration. | Any | Physiological and neuropsychological | None | Reported no conflict of interest Ministerio de Trabajo of Colombia, Fondo de Riesgos Laborales |
| (Picton et al., 2020) | Adults who have a diagnosis of a mental illness, live in the community, and have been referred to a Therapeutic Recreation program by mental health services | Nature-Based Therapeutic Recreation | No restriction | No restriction | N/A | Unreported |
| (Pomfret et al., 2023) | No restriction | Outdoor Activity / Adventures | No restriction | Wellbeing, friluftsliv, flow, peak experience, optimal experience | N/A | Reported no conflict of interest |
| (Quan et al., 2020) | Older Adults | Forest Therapy | Randomised Control Trial | No restriction | N/A | Reported no conflict of interest |
| (Rosa et al., 2023) | Studies with humans at any age, healthy or unhealthy | Nature-Based Adventure | No restriction | Studies that assess depression using a measure designed to measure depression. | Physical Activity, Satisfaction of basic psychological needs (autonomy, competence, and relatedness), nature connectedness, counselling and positive social interactions, confronting challenges, Participant Motivation | Reported no conflict of interest Spanish Ministry of Science, Education and Universities Coordenação de Aperfeiçoamento de Pessoal de Nível Superior—Brasil |
| (Rueff & Reese, 2023) | No restriction | Psychotherapy: Cognitive Behavioural Therapy, Group Cognitive Behavioral Therapy, Eco-therapy: Green exercise, Green care, Nature therapy, Adventure therapy, Psychotherapy in nature, Farm therapy, Forest bathing, “Shinrin-yoku” | No restriction | Self-assessment through questionnaires (DASS-21, BDIII, etc.) that capture anxiety | N/A | Reported no conflict of interest |
| (Shanahan et al., 2009) | Youth with Traumatic Brain Injury | Wilderness Adventure Therapy | N/A | Cognitive Rehabilitation | N/A | Reported no conflict of interest |
| (Siah et al., 2023) | No restriction | Forest Bathing (any type of activities conducted physically in the forest or natural settings) | No restriction | No restriction | N/A | Reported no conflict of interest Community Foundation of Singapore: Mind the Gap 200 – Mental Health Fund |
| (Smith et al., 2024) | Adults with persistent pain | Mindfulness Nature-Based Mindfulness Natural Environment | N/A | N/A | None | Reported no specific funding or conflict of interest |
| (Struthers et al., 2024) | Any human participant with a physical health condition | Outdoor, NBIs instructed by a health provider (e.g., physicians, physical therapists) or researcher | Any comparison including no intervention, other outdoor NBIs, indoor NBIs, or an intervention taking place in a non-nature-based setting | Physical health outcomes and/or biomarkers related to physical health conditions | None | Reported no specific funding or conflict of interest |
| (Taylor et al., 2022) | People with long-term physical health conditions limited to cardiovascular disease, stroke, lung and liver disease, type II diabetes and chronic kidney disease | Nature-, green- or outdoor animal-based interventions | Treatment as usual, urban environments or no comparison | Evaluation of effectiveness, as measured by improvements in psychological wellbeing and/or quality of life (QoL) | N/A | Reported no conflict of interest Stoneygate Trust National Institute for Health Research (NIHR) Leicester Biomedical Research Centre |
| (Trøstrup et al., 2019) | Inpatient, Outpatient, Patient | Nature-Based, Gardening, Horticulture, Wilderness | No restriction | Psychosocial, Rehabilitation, Restoration, Recovery, Wellbeing, Psychology, Quality of Life. Life Satisfaction, Recreation, Self, personal development, personal growth, grief, crisis, Happiness, Anxiety, Depression, psychological stress, mental stress, Suffering, Illness, Spirit, Hope, Meaning, Resilience, Burn-out, Cope, Coping, mental, health, mindful, emotion | N/A | Reported no conflict of interest |
| (Vella-Brodrick & Gilowska, 2022) | School Children | Nature Exposure | Urban, Classroom | Enhanced Cognitive Functioning | Social Aspects Physical Activity Age Study Duration | Open Access funding enabled and organized by CAUL and its Member Institutions |
| (Walker-Mao et al., 2024) | Postpartum women and parents | Horticultural Therapy Nature Exposure | N/A | Cognitive Function and Mental Health (mood, depression, anxiety) Loneliness, Time spent in Nature, Nature Visits | None | Reported no specific funding or conflict of interest |
| (Wang et al., 2022) | 60+ years of age | Any form of Horticultural Therapy involving therapeutic gardens, allotment gardening, and home gardening | Traditional activities, waiting list, non-gardener, not using any kind of Horticultural Therapy, or placebo. | Quality of life, Physical function, body mass index, mood related patient reported outcomes, blood pressure, and participant immunity | None | Reported no conflict of interest Scientific Research Project of Shanxi Provincial Health Commission Youth Basic Research Program of Shanxi Province |
| (Wang et al., 2024) | Participants from industrialized societies within an urban context | Physical or virtual intervention that involves nature | Control group required | Studies measuring depression, anxiety, and other psychopathological aspects | None | Reported no specific funding or conflict of interest |
| (Wen et al., 2019) | Any human participant | Forest bathing | N/A | Health | N/A | Reported no conflict of interest  National Natural Science Foundation of China |
| (Wen et al., 2024) | Patients | Natural environments or highly realistic computer- generated natural scenes; Immersive or semi-immersive VR methods | Any | Physical or psychological | None | Reported no specific funding or conflict of interest |
| (Yeo et al., 2020) | Adults Aged 60+ | Any form of real or simulated indoor nature exposure (excluding window views of outdoor nature, and animal-assisted therapy) | Non-nature interventions (e.g., music groups) or no-intervention (i.e., “usual care”) control groups. Also accepted single group before-after-after (“pre/post”) studies | Any health or wellbeing outcome | Duration Setting Dementia Shared/Group Experiences/Social Interaction Acquiring Knowledge/Skills/Lifelong Learning Having Autonomy/Responsibility | Reported no conflict of interest Supported by The College of Medicine and Health, University of Exeter The BlueHealth project which received funding from the European Union’s Horizon 2020 Research and Innovation Programme The National Institute for Health Research (NIHR) Collaboration for Leadership in Applied Health Research and Care South West Peninsula at the Royal Devon and Exeter NHS Foundation Trust |
| (Yun et al., 2024) | Individuals aged ≥ 60 years | Horticultural Therapy | A group of elders who participate in traditional activities or therapies, receive regular care or exercise, or attend educational classes, but do not participate in any gardening activities. | Changes in physical or mental health or changes in lifestyle | None | Reported no conflict of interest National Natural Science Foundation of China (No.32001367) and Open Fund of innova- tion institute for Sustainable Maritime Architecture Research and Technology (iSMART), Qingdao University of Technology (No. C2020-037) |
| (Zhao et al., 2022) | Persons with Disabilities | Horticultural Therapy (cultivation, topiary, trimming, weeding, floriculture or viewing the natural picture) | Nonpharmacological interventions and usual dementia care | Total score of cognitive function, the total score of agitation, total score of engagement, the total score of positive emotion. | None | Reported no conflict of interest Beijing Municipal Science & Technology Commission |
